# Long-Term Impact of Neonatal Acute Kidney Injury on Renal Function in Children Born Preterm: A Follow-Up Study

**DOI:** 10.3390/children12081018

**Published:** 2025-08-01

**Authors:** Tuğba Barsan Kaya, Özge Aydemir, Ozge Surmeli Onay, Evin Kocaturk, Çiğdem Öztunalı, Aslı Kavaz Tufan, Nuran Cetin, Özkan Alataş, Ayşe Neslihan Tekin

**Affiliations:** 1 Division of Neonatology, Department of Pediatrics, Faculty of Medicine, Eskisehir Osmangazi University, Eskisehir 26040, Turkey; oaydemir@ogu.edu.tr (Ö.A.); oonay@ogu.edu.tr (O.S.O.); ntekin@ogu.edu.tr (A.N.T.); 2 Department of Medical Biochemistry, Faculty of Medicine, Eskisehir Osmangazi University, Eskisehir 26040, Turkey; ekocaturk@ogu.edu.tr (E.K.); oalatas@ogu.edu.tr (Ö.A.); 3 Department of Pediatric Radiology, Faculty of Medicine, Eskisehir Osmangazi University, Eskisehir 26040, Turkey; cigdem.oztunali@ogu.edu.tr; 4 Department of Pediatric Nephrology, Faculty of Medicine, Eskisehir Osmangazi University, Eskisehir 26040, Turkey; aslikavaz@ogu.edu.tr (A.K.T.); nucetin@ogu.edu.tr (N.C.)

**Keywords:** acute kidney injury, preterm infants, long-term renal functions, kidney injury biomarkers, ambulatory blood pressure monitoring, chronic kidney disease

## Abstract

**Background and Objectives:** The long-term renal and cardiovascular effects of neonatal acute kidney injury (AKI) in preterm infants remain unclear. This study investigated whether neonatal AKI leads to persistent subclinical kidney injury and blood pressure changes in school-aged children born preterm. **Methods:** In this prospective cohort, preterm-born children (≤35 weeks’ gestation) with (n = 19) and without (n = 38) neonatal AKI were evaluated at 7–12 years. A term-born control group (n = 44) was included for biomarker comparison. Assessments included perinatal data, anthropometry, office and ambulatory blood pressure monitoring (ABPM), and renal ultrasonography. Kidney function was evaluated using serum creatinine (sCr), cystatin C, and estimated glomerular filtration rate (eGFR). Tubular injury was assessed using urinary kidney injury molecule-1/Cr (KIM-1/Cr), neutrophil gelatinase-associated lipocalin/Cr (NGAL/Cr), and trefoil factor 3/Cr (TFF3/Cr) ratios, as well as serum TFF3. **Results:** Conventional kidney function markers were similar among groups. However, the AKI group had higher serum cystatin C, lower cystatin C–based eGFR, and elevated urinary KIM-1/Cr and NGAL/Cr compared to no-AKI and term controls. Serum TFF3 was also higher in the AKI group. ABPM revealed higher nocturnal systolic blood pressure and blood pressure load in the AKI group. Kidney size did not differ between preterm subgroups. **Conclusions:** Neonatal AKI in preterm infants is associated with subtle alterations and potential renal stress or injury at school age, detectable only with sensitive biomarkers and ABPM. Further prospective studies are needed to validate these biomarkers and determine their role in predicting long-term outcomes in preterm infants with neonatal AKI.

## 1. Introduction

Acute kidney injury (AKI) is a common complication in the neonatal intensive care unit (NICU), particularly among preterm and critically ill infants. According to the Worldwide Acute Kidney Injury Epidemiology in Neonates (AWAKEN) data, one episode of AKI was observed in 30% of critically ill neonates [1]. However, in preterm infants, this incidence has been reported to be as high as 48%. AKI, once considered fully reversible, is now known to increase the risk of chronic kidney disease (CKD) in children [2]. Despite normalization of serum creatinine (sCr) at discharge in many neonates with AKI, long-term renal consequences often go unrecognized [3,4]. However, there is still no consensus on structured long-term follow-up protocols for neonates with AKI, particularly those born preterm. Current pediatric guidelines recommend routine blood pressure monitoring from age three, with ambulatory blood pressure monitoring (ABPM) for high-risk children; however, they do not specifically address neonatal AKI survivors [5]. It is unclear whether measuring albuminuria and the estimated glomerular filtration rate (eGFR) based on sCr capture the early kidney function decline in preterm-born patients, who often have a reduced number of nephrons and compensatory glomerular hypertrophy and hyperfiltration [6]. Recent studies highlight the value of novel biomarkers—kidney injury molecule-1 (KIM-1) [7,8,9], neutrophil gelatinase-associated lipocalin (NGAL) [10,11,12], and trefoil factor 3 (TFF3) [13,14]—for early detection of tubular injury and CKD risk.

This study investigates the long-term renal consequences of neonatal AKI in preterm-born children, comparing those with and without a history of AKI. We hypothesized that early-life AKI leads to persistent subclinical dysfunction, detectable by elevated levels of KIM-1, NGAL, and TFF3, as well as subtle abnormalities in renal function and ultrasonography. Understanding these long-term outcomes is crucial for developing evidence-based follow-up strategies for this vulnerable population.

## 2. Materials and Methods

### 2.1. Study Design and Ethical Approval

This single-center, prospective cohort study with retrospective baseline data was conducted at a university hospital between July 2022 and June 2024.

### 2.2. Study Population

The study included school-aged children (7–12 years old) who were born preterm (≤35 weeks of gestation) and admitted to the NICU between 2012 and 2015, as well as term-born controls (≥37 weeks’ gestation) who were appropriate for gestational age and had no known renal risk factors. Preterm children were identified from hospital records and invited for follow-up by phone; only those attending the visit and meeting eligibility criteria were enrolled. Controls were randomly selected from healthy attending child health supervision visits at our pediatric outpatient clinic, matched for age and sex, where possible, with no history of NICU admission or chronic illness. Exclusion criteria were major congenital anomalies or genetic syndromes affecting renal function, known renal disease or hypertension, and use of antihypertensive medication. Although the exclusion of children with a history of stage 3 AKI was not initially planned, most infants with stage 3 AKI could not be included in the study due to underlying renal anomalies, high neonatal mortality (mainly from asphyxia or septic shock), frequent hospitalizations, repeated exposure to nephrotoxic medications after discharge, or family refusal to participate in long-term follow-up.

### 2.3. Data Collection

#### 2.3.1. Neonatal Period

Retrospective data included perinatal characteristics, nephrotoxic drug exposure, and comorbidities [15]. Kidney-specific data during the NICU stay included the highest AKI stage reached, initial sCr measured at approximately 24 h of life, sCr at the end of the first week, peak sCr, and final sCr before discharge. AKI was classified using the modified Kidney Disease Improving Global Outcomes (KDIGOs) criteria, excluding urine output due to imprecise diaper-based monitoring [16] (see Table A1). GFR was estimated using the Schwartz formula (k = 0.33 for preterm infants) [17].

#### 2.3.2. School-Age Follow-Up Assessments

At follow-up, height and weight were measured to the nearest 0.1 cm and 0.1 kg using a wall-mounted stadiometer and a digital beam scale (Seca 767, Hamburg, Germany); body mass index (BMI) was calculated, and z-scores were determined using WHO reference data [18]. Blood pressure (BP) was measured per pediatric guidelines [19]. ABPM was performed using a validated oscillometric device (Mobil-O-Graph, IEM, Stolberg, Germany) on the non-dominant upper arm. Hypertension was defined according to the AHA guidelines [20]. Blood pressure load was calculated as the percentage of readings above the 95th percentile for systolic and diastolic values during daytime and nighttime. Office BP was measured in all groups, whereas ABPM was performed only in preterm-born children.

#### 2.3.3. Clinical and Laboratory Assessments at School Age

Blood samples were analyzed for sCr, cystatin C, and TFF3. Morning spot urine samples were collected during a dedicated study visit at our outpatient clinic and analyzed for renal injury markers, including NGAL, TFF3, KIM-1, urinary creatinine (uCr), and microalbumin. Routine laboratory measurements, including sCr, complete urinalysis, uCr, and microalbumin levels, were performed at the time of clinic admission. For biomarker analysis, blood and urine samples were centrifuged at 1000× *g* for 10 min and stored at −80 °C until analysis.

Serum and urine Cr levels were measured by the Jaffe method, urine microalbumin was measured by immunoturbidimetry, and the urine α1-microglobulin/creatinine ratio was calculated.

Serum TFF3 and cystatin C, as well as urinary NGAL, TFF3, and KIM-1, were measured using commercial ELISA kits (Cloud-Clone Corp., Wuhan, China) according to the manufacturer’s instructions. Urinary creatinine was measured with a commercial kit (SunLong Biotech, Hangzhou, China). Specimens were diluted as needed before analysis. Biomarker concentrations were expressed as recommended by the manufacturers, and urinary NGAL, TFF3, and KIM-1 levels were normalized to uCr.

GFR was calculated using the Schwartz formula (k = 0.55 for children) and the cystatin C-based 2012 CKD-EPI equation [21].

#### 2.3.4. Ultrasonographic Assessment at School Age

Renal ultrasonography was performed in the preterm-born cohort by an experienced pediatric radiologist using a Samsung HS50 system with high-frequency linear and convex probes. Each child was evaluated in both supine and prone positions, and kidneys were imaged longitudinally through the hilum. At least three measurements of maximal renal length were obtained per position, with the largest value used for analysis. Relative kidney length was calculated as kidney length divided by body height, and Z-scores and percentiles were determined using normative data from Obrycki et al. [22].

### 2.4. Statistical Analysis

Power analysis based on cystatin C-derived GFR showed that 18 participants per group were needed for 95% power (α = 0.05, β = 0.05), while post-hoc analysis with the current sample size demonstrated > 99% power.

Statistical analyses were performed using IBM SPSS Statistics v25.0 (IBM Corp., Armonk, NY, USA). Normality was assessed with the Shapiro–Wilk test. Continuous variables are presented as mean ± SD or median (Q1–Q3), and categorical variables as frequencies and percentages. Group comparisons used the independent samples *t*-test or Mann–Whitney U test for continuous variables, and chi-square or Fisher’s exact test for categorical variables. For three-group comparisons (AKI, no-AKI, controls), Kruskal–Wallis or one-way ANOVA was used, with Bonferroni or Dunn’s post hoc tests as appropriate. Correlations between gestational age and biomarker levels were assessed using Spearman’s rank correlation. To assess the independent associations of AKI status, birthweight, and gestational age with biomarker levels, multivariate linear regression analyses were performed. A *p*-value < 0.05 was considered statistically significant.

## 3. Results

**Figure 1:**
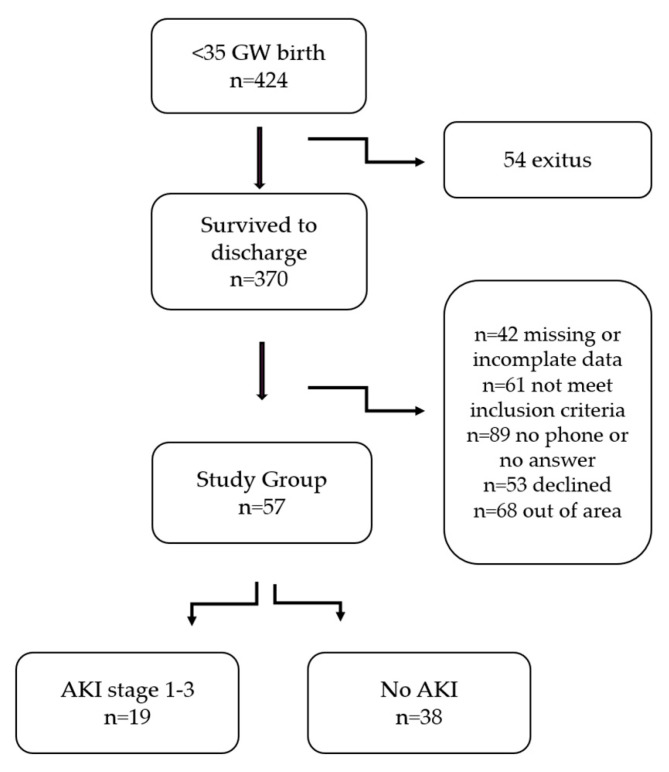
Flow chart of participant selection and study follow-up.

During the study period, 424 infants born at ≤35 weeks’ gestation were admitted to the NICU, and 54 (12.7%) of them died before discharge. After applying exclusion criteria and accounting for loss to follow-up, 57 preterm-born children were included. Additionally, 44 term-born children served as controls (Figure 1).

### 3.1. Clinical Demographics During NICU Hospitalization

Of the 57 preterm-born children, 19 (33.3%) experienced AKI (stage 1: n = 10; stage 2: n = 9), while 38 did not, and none required dialysis or other renal replacement therapy during the neonatal period. Infants with more severe AKI (stage 3) were not included in our study population, mainly due to high neonatal mortality and other exclusion criteria, as described in Section 2. Oligohydramnios and multiple gestation were more frequent in the AKI group, who also had lower gestational age, birth weight, and Apgar scores. SNAPPE II scores and rates of hypotension, early-onset sepsis, and BPD were higher in this group. Infants in the AKI group required longer invasive ventilation, parenteral nutrition, and hospitalization. They also had higher postmenstrual age and weight at discharge. They also received higher cumulative doses and/or longer durations of aminoglycosides, vancomycin, and caffeine. Initial sCr was similar between groups, but first week sCr, highest sCr during NICU stay, and lowest eGFR during NICU stay differed significantly. No significant differences were found in final sCr and eGFR at discharge. Clinical demographics are summarized in Table 1.

### 3.2. Anthropometric and Blood Pressure Findings at Follow-Up

At follow-up, age, weight, and BMI did not differ significantly among groups. Height was lowest in the no-AKI group, with a significant difference observed only when compared to controls; however, height z-scores were similar across all groups. Office BP measurements showed no significant intergroup differences (Table 2).

ABPM was initially planned for the preterm cohort. However, due to device unavailability or non-return, ABPM data were obtained from only 28 preterm subjects (8 AKI, 20 no-AKI). Notably, the AKI group exhibited significantly higher sleep systolic blood pressure (SBP) and mean blood pressure (MBP) than the no-AKI group (*p* < 0.05). Additionally, sleep-time SBP and diastolic blood pressure (DBP) load percentages were significantly higher in the AKI group (*p* = 0.04 and *p* = 0.02, respectively; Table 3). Among the analyzed patients, three had Stage 1 hypertension and four had elevated blood pressure; none were started on anti-hypertensive medication, and all were placed under close follow-up.

### 3.3. Renal Ultrasound Findings at Follow-Up

Renal ultrasonography was completed in 38 of 57 preterm children (AKI: n = 14; no-AKI: n = 24), as some families could not participate due to logistical or personal reasons. Comparisons of renal dimensions between the AKI and no-AKI groups revealed no statistically significant differences in kidney length, percentiles, or z-scores (Table 4).

### 3.4. Biomarkers of Renal Function and Injury in Comparison with the Term Cohort at Follow-Up

Renal function and injury biomarkers were compared among preterm-born children with and without a history of neonatal AKI and healthy term-born controls. No significant differences were observed in sCr, urine microalbumin-to-creatinine ratio, or protein-to-creatinine ratio among groups. Although sCr-based eGFR did not differ significantly, the AKI group had the lowest mean eGFR with a marginally significant difference versus controls (pa-c = 0.048) (Table 5).

Cystatin C was significantly higher in the AKI group than controls (*p* < 0.05), but not versus the no-AKI group (*p* = 0.10). Cystatin C–based eGFR was significantly lower in the AKI group than in both other groups (*p* = 0.002) (Table 5).

Serum TFF3 was significantly elevated in both the AKI and no-AKI groups compared to controls (*p* < 0.05 and *p* = 0.04, respectively), but not between the AKI and no-AKI groups (*p* = 0.07) (Table 5).

Urinary NGAL/creatinine and KIM-1/creatinine ratios were significantly higher in the AKI group than in both other groups, with no significant differences between no-AKI and controls. Urinary TFF3/creatinine ratios did not differ among groups (*p* = 0.63) (Table 5).

Comparing AKI severity, urine KIM-1/creatinine [median (IQR): 0.28 (0.11–0.48) vs. 0.62 (0.42–1.10), *p* = 0.013] and urine NGAL/creatinine [3.15 (2.15–6.67) vs. 10.8 (3.35–22.36), *p* = 0.030] were significantly higher in Stage 2 than Stage 1; urine TFF3/creatinine [7.77 (3.3–10.9) vs. 10.4 (6.8–16.1), *p* = 0.182] and serum TFF3 [1.11 (1.03–1.26) vs. 1.02 (0.79–1.26), *p* = 0.367] did not differ.

No significant correlations were found between gestational age and any biomarker, including sTFF3, NGAL/creatinine, uTFF3/creatinine, uKIM-1/creatinine, and cystatin C (for AKI group, respectively: ρ = 0.013, *p* = 0.957; ρ = 0.098, *p* = 0.690; ρ = 0.061, *p* = 0.804; ρ = 0.083, *p* = 0.735; ρ = –0.425, *p* = 0.070).

In multivariate linear regression analyses, only AKI status was independently associated with higher serum TFF3, urinary NGAL/creatinine, and urinary KIM-1/creatinine levels, while birthweight and gestational age were not significant predictors for any of these biomarkers. None of the variables were significantly associated with urinary TFF3/creatinine ratio (Table 6).

## 4. Discussion

Our study found no differences in conventional renal function markers (sCr, creatinine-based eGFR, and urinary protein excretion) among school-aged preterm-born children, with and without neonatal AKI, and term-born controls. However, preterm-born children with neonatal AKI, unlike both preterm peers without AKI and term-born controls, demonstrated lower cystatin C-based eGFR, higher nocturnal SBP, and increased blood pressure load in ABPM, elevated urinary KIM-1/creatinine and NGAL/creatinine levels, and higher serum TFF3. These findings suggest that, even in the presence of normal conventional markers, children with a history of neonatal AKI following preterm birth may exhibit subtle, long-term renal alterations and early signs of cardiovascular risk.

Long-term renal outcomes of premature-born children with neonatal AKI remain heterogeneous across studies, largely due to differences in study populations and diagnostic criteria. For instance, Abitbol et al. [23] reported a high rate of renal dysfunction in extremely low birth weight (ELBW) infants, possibly due to more severe baseline characteristics and stricter AKI definitions. Conversely, the IRENEO study found no differences in conventional renal function markers between preterm children with and without neonatal AKI, although both groups, especially those with very low birth weight (VLBW), frequently exhibited reduced renal volume and lower eGFR, suggesting early nephron deficit [4]. The FANCY study further demonstrated that children with neonatal AKI and VLBW had a higher risk of renal dysfunction in childhood, particularly with sensitive biomarkers such as cystatin C-based eGFR and proteinuria, which may detect subclinical renal injury not captured by conventional markers [24]. Similarly, we found no significant differences in sCr, creatinine-based GFR, or proteinuria among the AKI, non-AKI, or control groups, consistent with IRENEO and FANCY [3,24]. However, cystatin C-based GFR analyses revealed significant reductions in the AKI group, in parallel with the FANCY study [24]. These findings suggest that there may be a “silent period” during which conventional methods fail to detect early renal impairment, and more sensitive biomarkers could identify subtle renal alterations earlier. Reviews have emphasized that renal function impairments in preterm-born individuals often become more apparent as they grow older, indicating that early assessments may not be sufficient to detect subtle deficits [25]. Importantly, as highlighted in a recent systematic review [25], the lack of significant differences in conventional renal outcomes between groups in many studies may be partly due to the low number or exclusion of patients with severe (stage 3) AKI. This limitation, which also applies to our cohort, may have contributed to the absence of marked differences in conventional biomarkers, growth, and kidney size. Nevertheless, the observation that even children with mild to moderate (stage 1–2) AKI may exhibit subtle long-term alterations in renal function underscores the importance of close follow-up and supports the view that prematurity and any degree of neonatal AKI can have lasting renal consequences.

Proteomic biomarkers have the potential to transform kidney disease management by enabling earlier and more precise detection of renal injury. Consistent with previous reports highlighting KIM-1 as a sensitive marker of tubular injury [26], we found that urinary KIM-1/creatinine was significantly elevated in preterm-born children with a history of neonatal AKI compared to both other groups. Recent pediatric CKD cohort studies have also emphasized the prognostic value of KIM-1. For instance, Greenberg et al. [27] demonstrated that a two-fold increase in urinary KIM-1 was independently associated with a 50% decline in eGFR or progression to kidney failure in the CKiD study. Similarly, Szumińska et al. [28] confirmed urinary KIM-1 as a marker of tubulointerstitial injury and disease progression, particularly in glomerular and interstitial pathologies.

NGAL, which is rapidly upregulated in response to tubular injury, has also been suggested as a sensitive marker of tubulointerstitial damage and a potential predictor of CKD progression [29]. NGAL is one of the most extensively studied biomarkers for early kidney injury detection, with numerous studies demonstrating its sensitivity to tubular damage and its potential role in predicting both acute and chronic renal outcomes in various populations, including neonates and preterm-born children [30,31,32]. In our cohort, urinary NGAL/creatinine was significantly higher in preterm-born children with neonatal AKI, but not in the no-AKI or control groups, aligning with the findings of Atzori et al. [30], who reported elevated urinary NGAL in adults born with ELBW. However, Staub et al. [33] reported no significant differences in urinary NGAL between preterm and term adolescents, although serum NGAL was higher in preterm-born individuals with elevated blood pressure. Notably, previous studies did not stratify by neonatal AKI status, which may explain conflicting results.

In our study, biomarker levels at school age were not significantly associated with gestational age in either group. Interestingly, urine KIM-1/creatinine and NGAL/creatinine levels tended to be higher in Stage 2 compared to Stage 1 AKI, potentially suggesting an association between AKI severity and tubular injury. However, all AKI cases in our cohort were stage 1 or 2, and the absence of severe (stage 3) AKI may have limited our ability to detect more pronounced biomarker elevations. Additionally, differences in gestational age between preterm subgroups could confound associations, though our analysis found no significant relationship between gestational age and kidney injury biomarkers at school age, consistent with previous studies [34]. These findings suggest that elevated urinary KIM-1 and NGAL may signal subtle or early changes in renal status in preterm-born children with neonatal AKI, even when conventional markers are normal. Importantly, our results indicate that a history of neonatal AKI may represent an independent risk factor for later renal alterations in preterm-born children, beyond the effects of prematurity alone. However, whether these biomarker elevations represent persistent injury or transient adaptation remains unclear, and their clinical significance requires further investigation through long-term, longitudinal studies.

TFF3, an emerging biomarker linked to epithelial repair, has previously been studied in neonates mainly in the context of nephrogenesis and postnatal adaptation. In our study, we assessed both serum and urinary TFF3 in school-aged preterm-born children, stratified by neonatal AKI history. We observed significantly elevated serum TFF3 in the AKI group, while urinary TFF3 levels did not differ among groups. Furthermore, neither serum nor urinary TFF3 levels differed significantly between Stage 1 and Stage 2 AKI. In the literature, Kamianowska et al. [35] and Corrêa et al. [36] reported higher urinary TFF3 in preterm neonates, which was thought to reflect developmental processes rather than injury. However, no studies have specifically investigated the relationship between neonatal AKI or its long-term effects and TFF3. In contrast, larger series in adults have demonstrated that both serum and urine TFF3 levels increase with the progression of kidney injury [13,37], and elevated serum TFF3 in adults has also been associated with non-renal conditions such as inflammation, malignancy, and gastrointestinal disorders [13]. This discrepancy suggests that elevated serum TFF3 in our cohort may not be directly attributable to renal injury and highlights the need for further research to clarify the clinical significance and specificity of TFF3 as a biomarker in pediatric populations.

### 4.1. Blood Pressure

Recent meta-analyses indicate that preterm-born individuals are at increased risk for long-term hypertension and altered circadian blood pressure rhythms [38]. In our study, preterm-born children with a history of neonatal AKI had significantly higher sleep SBP, sleep MBP, and systolic and diastolic BP load on ABPM compared to those without AKI. These findings suggest an association between neonatal AKI and altered blood pressure patterns in preterm children, potentially reflecting early vascular change. Although current guidelines recognize prematurity as a hypertension risk factor, they do not specifically address neonatal AKI in surveillance strategies [20]. Our results support a more refined risk stratification that incorporates both gestational age and early kidney injury when planning long-term follow-up. While the absence of ABPM data from controls limits comparison, the observed differences between preterm-born children with and without neonatal AKI provide valuable insights into the cardiovascular impact of neonatal renal injury.

### 4.2. Ultrasound Findings

In our study, kidney length, relative size, and z-scores did not differ significantly between preterm children with and without neonatal AKI. This is consistent with the IRENEO study, which found lower renal volumes in children with a history of neonatal AKI compared to those without, but this difference was not significant after adjusting for birth weight [3]. Similarly, the FANCY study reported no differences in kidney size despite higher rates of renal dysfunction in the AKI group based on cystatin C and proteinuria [24]. These findings indicate that while functional impairment may persist after AKI, structural changes are often subtle and may not be detected by ultrasound, highlighting the need for further research.

### 4.3. Limitations and Strengths

This study has several limitations. Being conducted at a single center with a relatively small sample size may limit the generalizability of our findings and highlights the need for validation in larger, multicenter cohorts. The absence of a term-born control group for kidney size and ABPM restricted full comparative analysis. AKI was defined using modified KDIGO criteria; however, retrospective data collection precluded accurate urine output assessment, so classification relied solely on serum creatinine changes. All AKI cases were stage 1 or 2; infants with severe (stage 3) AKI were excluded due to high neonatal mortality, family unwillingness to participate, and complex medication regimens. Additionally, differences in gestational age between preterm subgroups could confound associations, though our analysis found no significant relationship between gestational age and kidney injury biomarkers at school age, consistent with previous studies [34].

Despite these limitations, the study’s prospective, longitudinal design—tracking children from the neonatal period to school age—enables a thorough evaluation of the long-term effects of neonatal AKI in preterm-born children. Including both preterm children with and without AKI, as well as a term-born control group, allows for meaningful comparisons and clarifies the specific impact of neonatal AKI beyond prematurity alone. The use of both traditional and novel biomarkers (KIM-1, NGAL, TFF3) provides a sensitive and comprehensive assessment of kidney health, while ABPM offers valuable insights into circadian blood pressure patterns. Our findings indicate that even mild to moderate neonatal AKI in preterm infants may have subtle but lasting effects on renal health, highlighting the need for long-term monitoring in this vulnerable population.

## 5. Conclusions

This study demonstrates that preterm-born children, especially those with neonatal AKI, may exhibit subtle alterations in renal function and blood pressure at school age, when conventional markers appear normal. Notably, while conventional markers did not show significant differences in kidney injury between groups, sensitive biomarkers such as urinary KIM-1 and NGAL, which are more extensively studied and robust in the literature, revealed subtle but significant renal alterations in children with a history of neonatal AKI. While sensitive biomarkers and ambulatory blood pressure monitoring may be valuable in long-term follow-up of preterm children with AKI, further prospective studies are needed to clarify their prognostic significance and determine whether such approaches improve clinical outcomes. Until then, clinicians should remain vigilant in monitoring preterm-born children with a history of AKI—even if the AKI episode was mild or moderate—as this group may benefit from closer long-term follow-up for renal function and blood pressure.

## Figures and Tables

**Table 1:** Preterm infants with and without neonatal acute kidney injury: perinatal and neonatal characteristics.

	AKI (n = 19)	No AKI (n = 38)	*p* Value
Perinatal Factors			
Maternal age, year **	28 (23–32)	32 (25–37)	0.20
Multiple birth, n (%)	17 (44.7)	5 (26.3)	0.03
Maternal hypertension, n (%)	7 (38.9)	15 (39.5)	0.96
Betamethasone (complete course)	13 (68.4)	22 (57.9	0.56
Maternal diabetes, n (%)	2 (10.5)	9 (23.7)	0.23
PPROM, n (%)	1 (5.3)	4 (10.5)	0.50
Oligohydramnios, n (%)	11 (57.9)	6 (15.8)	0.002
Cesarean section, n (%)	17 (89.5)	34 (89.5)	1
Neonatal Factors			
Gestational age **	30 (28–33)	32 (31–33)	0.01
Birth weight (g) **	1335 (1015–1657)	1615 (1318–1920)	0.003
Birth weight percentile, % *	40.0 ± 6.2	47.2 ± 4.8	0.38
IUGR, n (%)	10 (52.6)	16 (42.1)	0.31
Male, n (%)	10 (52.6)	18 (47.4)	0.78
Apgar score (1 min) **	7 (4–8)	8.5 (7–9)	0.007
Apgar score (5 min) **	8.5 (7–10)	10 (8–10)	0.04
Ph < 7.2, n (%)	4 (21.1)	1 (2.9)	0.04
BE mmol/L *	7.5 ± 0.9	7,7 ± 0.6	0.93
Lactate, mmol/L *	3.7 ± 0.4	3.0 ± 0.2	0.08
SNAPPE II score **	34 (7–61)	5 (2.5–21)	<0.001
Invasive mechanical ventilation, days **	3 (0.5–11)	0 (0–1)	
Hypotension, n (%)	9 (47.4)	8 (21.1)	0.04
UVC, n (%)	13 (68.4)	18 (47.4)	0.13
UAC, n (%)	4 (21.1)	0	
Parenteral nutrition, days **	28 (15–51)	14 (5–23)	<0.001
Early sepsis, n (%)	7 (36.8)	2 (5.3)	0.004
Treated PDA, n (%)	5 (26.3)	4 (10.5)	0.14
NEC ≥ stage 2, n (%)	3 (15.8)	0	
BPD, n (%)	12 (63.2)	8 (22.9)	0.003
IVH grade 3 or 4, n (%)	3 (15.8)	0	
Length of hospitalization **	53 (36–66)	32 (20–47)	<0.001
Postmenstrual age at discharge, week **	38 (37–39)	37 (35–38)	<0.001
Weight at discharge, g **	2500 (2155–2715)	2300 (2090–2440)	0.05
Weight percentile at discharge, % **	10 (1.5–18.5)	11 (1–35)	0.19
Medications in NICU			
Ibuprofen, mg/kg **	20 (15–20)	20 (0–20)	0.32
Aminoglycoside, days *	17 ± 4.4	9.1 ± 2.2	0.002
Vancomycin, days **	11.5 (6–19)	0 (0–14)	0.01
Caffeine, days *	28.6 ± 7.9	19.4 ± 6.7	0.014
Kidney history in NICU			
Inıtial sCr, mg/dL **	0.62 (0.40–0.88)	0.72 (0.47–0.84)	0.71
1st week sCr, mg/dL *	0.60 ± 0.06	0.44 ± 0.03	0.01
1st week eGFR, mL/min/1.73 m^2^ **	22.9 (16.2–28.2)	29.0 (21.6–43.2)	0.006
Highest sCre during NICU stay, mg/dL **	0.86 (0.64–1.5)	0.40 (0.23–0.56)	0.006
Lowest eGFR during NICU stay, mL/min/1.73 m^2^ **	24.2 (18.4–30.2)	34.6 (23.8–49.9)	0.003
sCR at discharge, mg/dL **	0.3 (0.19–0.38)	0.26 (0.2–0.33)	0.42
eGFR at discharge, mL/min/1.73 m^2^ **	50.0 (37.3–89.6)	59.6 (49.5–87.4)	0.17

Values are presented as mean ± SD *, median (Q1–Q3) **, or n (%). Abbreviations: AKI, acute kidney injury; BE, base excess; BPD, bronchopulmonary dysplasia; C/S, cesarean section; sCre, serum creatinine; GFR, glomerular filtration rate; IVH, intraventricular hemorrhage; IUGR, intrauterine growth restriction; NEC, necrotizing enterocolitis; PDA, patent ductus arteriosus; PPROM, preterm premature rupture of membranes; SNAPPE-II, Score for Neonatal Acute Physiology with Perinatal Extension II; UAC, umbilical arterial catheter; UVC, umbilical venous catheter. Birth weight percentile was calculated according to gestational age using the Fenton growth chart. *p* < 0.05 was considered statistically significant.

**Table 2:** Anthropometry and blood pressure measurements among the groups.

At the Time of Follow-Up (7–12 Years of Age)	AKI ^a^ (n = 19)	No-AKI ^b^ (n = 38)	Controls ^c^ (n = 44)	*p* Value
Age (month) *	105 ± 2	97 ± 1	107 ± 4	0.06
Male, n (%)	10 (52.6)	18 (45)	28 (63.6)	0.22
Height (cm) *	129 ± 2.2	125.5 ± 1.0	132.0 ± 2.0	0.02 p^a-b^: 0.33 p^a-c^: 0.61 p^b-c^: 0.01
Height z-score **	129 (121–136)	126 (122–130)	129.5 (122.2–141.5)	0.14
Weight (kg) **	26 (23–37)	25 (22.7–32.7)	27.2 (21.2–31.7)	0.77
Weight z-score **	0.04 (−0.78–1.42)	−0.10 (−0.83–1.12)	−0.38 (−0.81–0.42)	0.38
BMI (kg/m^2^) **	16.8 (14.1–19.8)	15.9 (14.6–19.2)	15.9 (14.4–21.8)	0.64
BMI z-score *	0.20 ± 0.35	0.22 ± 0.19	−0.27 ± 0.16	0.13
Blood pressure				
Systolic (mmHg) **	100 (95–108.5)	100 (95.7–107.5)	108 (100–115)	0.06
Diastolic (mmHg) **	60.5 (56–66)	60 (55–62.2)	60 (51–64)	0.48
Mean MP (mmHg) **	77 (73–82.7)	76 (70–79.2)	75 (70–82.6)	0.27

Values are presented as mean ± SD *, median (Q1–Q3) **, or n (%), as appropriate. Abbreviations: BMI, body mass index; DBP, diastolic blood pressure; MBP, mean blood pressure; SBP, systolic blood pressure. a: AKI group; b: no-AKI group; c: control group. Pairwise comparisons were adjusted as follows: a-b, AKI vs. no-AKI; a-c, AKI vs. control; b-c, no-AKI vs. control. *p* < 0.05 was considered statistically significant.

**Table 3:** Ambulatory blood pressure monitoring in preterm children with and without neonatal acute kidney injury.

At the Time of Follow-Up (7–12 Years of Age)	AKI (n = 8)	No-AKI (n = 20)	*p* Value
Ambulatory BP (mmHg)			
Awake SBP **	97.5(96–106)	99.5 (95–106)	0.80
Awake DBP *	61.3 ± 2.3	63.1 ± 1.3	0.64
Awake MBP *	79.3 ± 2.1	80.5 ± 1.2	0.56
Sleep SBP *	96.1 ± 2.4	93.6 ± 1.1	0.02
Sleep DBP *	55.3 ± 1.0	55.8 ± 1.1	0.20
Sleep MBP *	73.7 ± 1.4	73.0 ± 1.0	0.04
Awake SBP Load (%) **	12.5 (3.2–19)	5.5 (2–26.2)	0.42
Awake DBP Load (%) **	13.0 (0–19)	13.5 (6–30.7)	0.96
Sleep SBP Load (%) *	31.5 ± 8.2	15.7 ± 3.1	0.04
Sleep DBP Load (%) *	20.3 ± 5.4	15.6 ± 3.4	0.02

* Values are presented as mean ± SD; ** values are presented as median (Q1–Q3), as appropriate. Abbreviations: DBP, diastolic blood pressure; MBP, mean blood pressure; SBP, systolic blood pressure. Load percentages represent the proportion of readings exceeding the 95th percentile for age, sex, and height. *p* < 0.05 was considered statistically significant.

**Table 4:** Renal ultrasound findings in preterm children with and without neonatal acute kidney injury.

At the Time of Follow-Up	AKI (n = 14)	No-AKI (n = 24)	*p* Value
Kidney USG			
Left kidney length, cm	8.25 (7.72; 8.7)	8.4 (7.5; 9.2)	0.67
Relative LK Length, cm	6.69 (6.21; 7.07)	6.45 (5.84; 6.78)	0.19
Left kidney length percentile, mm	25 (4.5; 42)	19.5 (4.2; 30.7)	0.72
Left kidney length z score	−0.67(−1.7; −0.2)	−0.86 (−1.77; −0.49)	0.72
Right Kidney length, cm	8.2 (7.5–8.85)	8.45 (8.17–9.2)	0.19
Relative RK Length, cm	6.57 (6.07; 7.27)	6.55 (6.07; 7.27)	0.63
Right kidney lenght percentile, mm	21(10.2; 46.5)	11.5 (1.2; 34.7)	0.23
Right kidney lenght z score	−0.60 ± 0.29	−1.05 ± 0.29	0.14
Mean kidney length/body length cm	0.61 (0.59; 0.68)	0.64 (0.58; 0.67)	0.43

Values are presented as mean ± SD, or median (Q1–Q3) as appropriate.

**Table 5:** Kidney function and injury biomarkers at school-age follow-up in preterm-born children with and without neonatal AKI versus term controls.

	AKI ^a^ (n = 19)	No AKI ^b^ (n = 38)	Controls ^c^ (n = 44)	*p* Value
Serum Cr, mg/dL **	0.52 (0.47–0.56)	0.47 (0.43–0.50)	0.51 (0.48–0.57)	0.17
eGFR, mL/min/1.73 m^2^ *	118.12 ± 3.53	120.0 ± 2.53	128.8 ± 2.26	0.61
Microalbumin/Cr, mg/mg **	10.48 (5.2–18.3)	5.7 (4.1–10.1)	6.5 (4.6–8.7)	0.14
Protein/Cr, mg/g *	134.7 ± 9.7	129.6 ± 4.9	147.5 ± 7.8	0.50
Cystatin C, serum, mg/L **	1.16 (0.74–2.24)	0.95 (0.54–2.58)	0.78 (0.52–1.05)	0.04 p^a-b^: 0.10 p^a-c^: <0.001 p^b-c^: 0.10
eGFR (CysC), mL/min/1.73 m^2^ *	90 ± 9.7	98 ± 9.1	106 ± 6.7	0.002 p^a-b^: 0.04 p^a-c^: <0.001 p^b-c^: 0.21
Serum TFF3, ng/mL *	1.05 ± 0.05	0.88 ± 0.04	0.77 ± 0.04	<0.001 p^a-b^: 0.07 p^a-c^: <0.001 p^b-c^: 0.04
Urinary NGAL/Cr, ng/mg Cr **	0.36 (0.22–1.15)	0.07 (0.02–0.18)	0.06 (0.03–0.14)	<0.001 p^a-b^: <0.001 p^a-c^: <0.001 p^b-c^: 0.94
Urinary TFF3/Cr, ng/mg **	9.4 (4.0–12.48)	7.0 (4.5–11.4)	8.1 (5.3–12.8)	0.63
Urinary KIM1/Cr, ng/mg Cr **	0.42 (0.22–0.76)	0.24 (0.19–0.28)	0.21 (0.13–0.42)	0.007 p^a-b^: 0.006 p^a-c^: 0.003 p^b-c^: 0.82

Values are presented as mean ± SD *, median (Q1–Q3) **, or n (%), as appropriate. Serum creatinine and cystatin C levels were used to estimate glomerular filtration rate (eGFR and GFRCysC, respectively; units: mL/min/1.73 m^2^). All urinary biomarkers are normalized to urinary creatinine. a: AKI group; b: no-AKI group; c: control group. Pairwise comparisons were adjusted as follows: a-b, AKI vs. no-AKI; a-c, AKI vs. control; b-c, no-AKI vs. control. *p* < 0.05 was considered statistically significant.

**Table 6:** Multivariate Linear Regression Analysis for Biomarker Levels.

Biomarker	Predictor	B (SE)	*p*-Value
uNGAL/creatinine	AKI	7.537 (1.553)	<0.001
	Gestational Age	0.259 (0.414)	0.534
	Birth weight	0.000 (0.002)	0.833
uKIM-1/creatinine	AKI	0.003 (0.001)	0.012
	Gestational Age	0.000 (0.000)	0.209
	Birth weight	−0.000001 (0.000)	0.491
uTFF3/creatinine	AKI	−0.004 (0.024)	0.873
	Gestational Age	0.010 (0.006)	0.143
	Birth weight	−0.00042(0.000)	0.144
sTFF3	AKI	0.173 (0.075)	0.024
	Gestational Age	0.022 (0.019)	0.259
	Birth weight	−0.000081 (0.000)	0.355

Abbreviations: AKI, acute kidney injury; B, unstandardized regression coefficient; SE, standard error; uNGAL/creatinine, urinary neutrophil gelatinase-associated lipocalin-to-creatinine ratio; uKIM-1/creatinine, urinary kidney injury molecule-1-to-creatinine ratio; uTFF3/creatinine, urinary trefoil-factor-3-to-creatinine ratio; sTFF3, serum trefoil factor 3. *p* < 0.05 indicates statistical significance.

## Data Availability

The datasets generated and/or analyzed during the current study are available from the corresponding author on reasonable request. The informed consent forms signed by participants did not include permission for unrestricted public data sharing, and the Ethical Review Board approval for this study included restrictions on public data sharing to protect participant confidentiality. Therefore, the data are not publicly available. However, researchers who meet the criteria for access to confidential data can obtain the data from the corresponding author.

## Abbreviations

The following abbreviations are used in this manuscript:

ABPM	Ambulatory Blood Pressure Monitoring
AKI	Acute Kidney Injury
AHA	American Heart Association
BPD	Bronchopulmonary Dysplasia
BMI	BMI Body Mass Index
BP	BP Blood Pressure
CKD	CKD Chronic Kidney Disease
Cr	Cr Creatinine
DBP	DBP Diastolic Blood Pressure
eGFR	eGFR Estimated Glomerular Filtration Rate
ELISA	ELISA Enzyme-Linked Immunosorbent Assay
ELBW	ELBW Extremely Low Birth Weight
IVH	IVH Intraventricular Hemorrhage
KIM-1	KIM-1 Kidney Injury Molecule-1
MBP	MBP Mean Blood Pressure
NEC	NEC Necrotizing Enterocolitis
NGAL	NGAL Neutrophil Gelatinase-Associated Lipocalin
NICU	NICU Neonatal Intensive Care Unit
PDA	PDA Patent Ductus Arteriosus
RDS	RDS Respiratory Distress Syndrome
SBP	SBP Systolic Blood Pressure
sCr	sCr Serum Creatinine
SNAPPE-II	SNAPPE-II Score for Neonatal Acute Physiology with Perinatal Extension-II
TFF3	TFF3 Trefoil Factor 3
uCr	uCr Urinary Creatinine
WHO	WHO World Health Organization
Z-score	Z-score Standard Deviation Score

## Appendix A

**Table A1:** Kidney Disease: Improving Global Outcomes (KDIGO) acute kidney injury (AKI) definition modified to exclude urine output.

Stage	Serum Creatinine
1	1.51.9 times baseline ≥0.3 mg/dL increase (48 h)
2	2.02.9 times baseline
3	3 times baseline ≥2.5 mg/d increase
